# Is there a relationship between surgical case volume and mortality in congenital heart disease services? A rapid evidence review

**DOI:** 10.1136/bmjopen-2015-009252

**Published:** 2015-12-18

**Authors:** L Preston, J Turner, A Booth, C O'Keeffe, F Campbell, A Jesurasa, K Cooper, E Goyder

**Affiliations:** School for Health and Related Research (ScHARR), University of Sheffield, Sheffield, UK

## Abstract

**Objective:**

To identify and synthesise the evidence on the relationship between surgical volume and patient outcomes for adults and children with congenital heart disease.

**Design:**

Evidence synthesis of interventional and observational studies.

**Data sources:**

MEDLINE, EMBASE, CINAHL, Cochrane Library and Web of Science (2009–2014) and citation searching, reference lists and recommendations from stakeholders (2003–2014) were used to identify evidence.

**Study selection:**

Quantitative observational and interventional studies with information on volume of surgical procedures and patient outcomes were included.

**Results:**

31 of the 34 papers identified (91.2%) included only paediatric patients. 25 (73.5%) investigated the relationship between volume and mortality, 7 (20.6%) mortality and other outcomes and 2 (5.9%) non-mortality outcomes only. 88.2% were from the US, 97% were multicentre studies and all were retrospective observational studies. 20 studies (58.8%) included all congenital heart disease conditions and 14 (41.2%) single conditions or procedures. No UK studies were identified. Most studies showed a relationship between volume and outcome but this relationship was not consistent. The relationship was stronger for single complex conditions or procedures. We found limited evidence about the impact of volume on non-mortality outcomes. A mixed picture emerged revealing a range of factors, in addition to volume, that influence outcome including condition severity, individual centre and surgeon effects and clinical advances over time.

**Conclusions:**

The heterogeneity of findings from observational studies suggests that, while a relationship between volume and outcome exists, this is unlikely to be a simple, independent and directly causal relationship. The effect of volume on outcome relative to the effect of other, as yet undetermined, health system factors remains a complex and unresolved research question.

**Strengths and limitations of this study**

- We conducted a rapid review in a very short timescale to identify key relevant evidence that could inform an ongoing service review.
- We used clear and reproducible methods for evidence searching, inclusion and exclusion criteria and data extraction.
- Time constraints means we could not search exhaustively and so some relevant evidence may have been missed.
- Detailed quality appraisal of individual included studies was replaced with a narrative summary of methodology and study design limitations.

## Introduction

An extensive evidence base supports an association between organisational factors and patient outcomes in elective surgery provision. The existence of a causal relationship between volume of activity and better patient outcomes is based on assumptions that more activity may be associated with better facilities, more experienced multidisciplinary teams and more experienced and specialist clinicians, rather than being simply attributable to increased workload.^1^ The volume and outcome association has been most extensively studied in the surgical specialities and for complex procedures where institutional and surgical experience and specialisation might be especially important in optimising outcomes.^2^ However, the underlying reasons for the observed associations between greater volumes of surgical activity and better outcomes for patients remain unclear and observed variations in outcomes, including mortality, remain unexplained.^3^

Evidence on the relationship between volume and outcome of surgery is dominated by studies evaluating the relationship with mortality. However, volume may exert important effects on other patient outcomes such as morbidity and quality of life as well as service consequences, such as length of stay in hospital and costs.

Services for congenital heart disease (CHD) have been subject to scrutiny for over a decade, in the UK and internationally. In 2012 a series of recommendations was made for the reconfiguration of cardiac services for children in England.^4^ However, the process for making these recommendations was challenged and, following a judicial review, service reconfiguration was not implemented and a new service review considering the whole lifetime pathway for CHD undertaken.

The objective of this evidence synthesis was to inform the service review by examining whether there is evidence for a relationship between institutional and individual surgeon surgical volume and patient outcomes in CHD services. Evidence for other explanatory variables, including organisational features and other outcomes (such as complications) were examined in the full review.^5^Here we summarise the evidence for the specific relationship between surgical volume and outcome.

## Methods

We undertook a keyword-based systematic literature search using a predefined protocol,^5^ (see online supplementary file 1) enhanced by supplementary search methods.^6^ Reporting follows the PRISMA guidelines.^7^ The review was completed within 3 months.

### Search strategy

Relevant articles were identified using a database search strategy adapted from an earlier systematic review completed in 2009 (see online supplementary file 2). Search terms included population, volume, other organisational factors (eg, proximity to other services such as intensive care) and patient-related outcomes. We conducted searches (January and March 2014) of MEDLINE, EMBASE, CINAHL, Cochrane Library and Web of Science for the years 2009–2014.

These formal keyword-based literature searches were supplemented by four additional search methods designed to identify additional studies not included in the earlier systematic review for the 11-year period 2003–2009. These included citation searching using key references; responses from patient and public groups and clinical experts following a call for evidence; scrutiny of the reference lists of included papers and examination of the reference lists of published reviews, guideline documents and reports.

### Selection criteria

Studies were eligible for inclusion if they reported an association between surgical volume (surgical unit or individual surgeon) and patient outcomes for children and/or adults undergoing treatment (surgical or interventional) for congenital heart disease. All types of patient-related outcomes (mortality, complications and quality of life) and health service outcomes (length of stay, costs) were eligible.

Studies eligible for inclusion were (1) observational studies and reports from trials. Qualitative or questionnaire-based studies were excluded. (2) Evidence from Organisation for Economic Cooperation and Development countries to ensure relative health system comparability to the UK. The review only included original research articles published in English and data from conference abstracts was excluded as these did not yield sufficient information. (3) Published in peer-reviewed journals to ensure that the evidence being synthesised had undergone methodological and expert scrutiny.

One author (LP) screened titles and abstracts using the inclusion and exclusion criteria. Second screening was undertaken for 10% of the references retrieved via the database searches by a second reviewer (KC) then five reviewers (JT, KC, AJ, CO and FC) screened the full text of any potentially relevant article. Each reviewer independently assessed the eligibility of each study, and the final list of included studies was agreed by consensus.

### Data extraction

Five reviewers (JT, KC, AJ, CO and FC) independently extracted information into a standardised data extraction form, piloted on three studies and refined accordingly. The data extraction form collected information on the characteristics of each study, the results as reported by the authors, risk adjustment undertaken and key messages (table 1). The primary outcome measure was mortality reported as ORs for the risk of dying or differences in percentage mortality rate for comparisons of low and high volume centres or surgeons. Any disagreements or challenges in data extraction were resolved with another member of the review team.

**Table 1:** Items included in data extraction

Study characteristics	Study findings
Study dates Study aim Study design Data source and type Study population Condition(s) Unit characteristics Intervention/procedure Definition of volume Outcomes measured Sample size Number of participants Number of events	Volume analysed as continuous or categorical variable Volume thresholds for categorical variables Covariates used in the analysis Crude associations of volume and outcome Adjusted associations of volume and outcome Linear or non-linear relationship Summary of main findings Summary of limitations identified by authors

### Quality assessment

As this was a rapid review we did not conduct a quality appraisal of individual included studies using a conventional quality assessment tool. Instead we used two complementary approaches to quality assessment to examine the collective contribution of the evidence base as a whole. First, we assessed the adequacy of the included evidence in addressing the aim of the research using a simple yes/no checklist for relevant factors including the study characteristics (eg, whether the study was single or multicentre or included more than one intervention/condition), the quality of the source data (eg, whether data collection was voluntary or mandatory) and the statistical analysis/adjustment (eg, whether the study adjusted for severity of condition and/or age). These relevance criteria for each included study are provided in online supplementary file 3. Second, we performed a study design level quality assessment to identify generic weaknesses. Similar study designs were coded with shared limitations. Judgements on quality were informed by limitations explicitly reported by study authors in the included studies. Assessment was undertaken by three authors (AB, JT and LP) and disagreements resolved by discussion.

### Data synthesis

We extracted and tabulated the study information and used this to produce a narrative synthesis. A meta-analysis was not feasible given the considerable heterogeneity in the design, methods and settings of the included studies.

## Results

### Study selection

**Figure 1:**
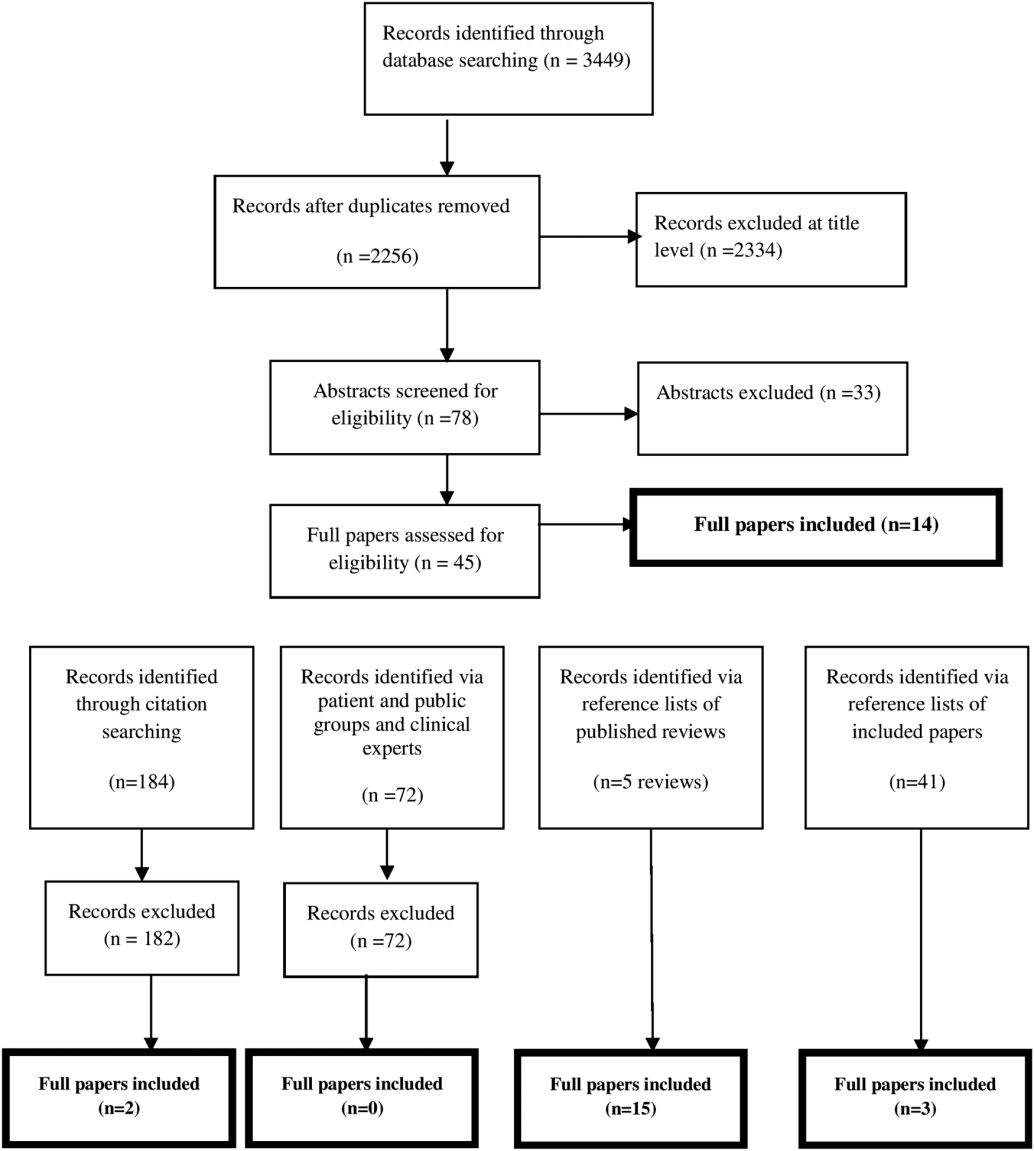
Modified PRISMA flow chart of study identification, listing reason for exclusion during review process.

The database search identified 2256 unique references of which 14 met inclusion criteria. An additional 20 papers were identified using additional non-database search methods giving a total of 34 included papers (figure 1).

### Characteristics of the reviewed studies

The characteristics of the 34 included papers from 34 individual studies are summarised in table 2.

**Table 2:** Characteristics of included studies

Study characteristics	Number (%)
Total number full papers included	34 (100)
Volume and outcome relationship all conditions	20 (58.8)
Volume and outcome relationship specific conditions/procedures	14 (41.2)
Country	
USA/Canada	30 (88.2)
Japan	2 (5.9)
Germany	1 (2.9)
Sweden	1 (2.9)
Centre type	
Multicentre	33 (97)
Single centre	1 (3)
Data sources	
Voluntary (STS-CHD, HCUP-KIDS, PCCC, UHC)	19 (55.9)
Involuntary/registry (PHIS, NIS, OSHPD, UNOS, Texas birth defects registry)	12 (35.3)
Study specific	3 (8.8)
Patient population	
All children (0–20)	19 (55.9)
Newborns and infants only	12 (35.3)
Adults	3 (8.8)
Outcomes measured	
Survival/mortality only	25 (73.5)
Survival/mortality and other outcomes	7 (20.6)
Other outcomes only	2 (5.9)
Design	
Retrospective cohort	28 (82.4)
Retrospective observational	1 (2.9)
Cross-sectional	3 (8.8)
Longitudinal	1 (2.9)
RCT (data source)	1 (2.9)

RCT, randomised controlled trial.

The majority of studies (88.3%) were conducted in the USA and most were multicentre (97%). No UK studies were identified. 31/34 included only paediatric patients. Thirty-one studies used routine data sets, including 19 voluntary clinical and/or administrative data sets, 12 using mandatory administrative data sets.^5^

Twenty-five of the 34 studies (73.5%) measured mortality or survival as the only outcome, eight studies measured mortality and other non-mortality outcomes including complications, length of stay in hospital, re-operation rates, length of ventilator treatment and time to extubation and costs. Two studies measured only non-mortality outcomes. Only 8 (25%) of the 32 studies reporting mortality measured this outcome postdischarge.

We have classified included studies into two groups—those where the primary objective was to explore the relationship between volume of service and outcomes for a range of CHD conditions (20/34) and those where the focus was on the relationship between volume and outcome for specific single conditions or procedures (14/34). For studies involving specific conditions or procedures these were mainly complex conditions such as hypoplastic left heart syndrome, transposition of great arteries and pulmonary atresia or procedures including Norwood Procedure, arterial switch operation and Blalock Taussig Shunt Procedure.^8–41^ Online supplementary table S3 provides a summary of the individual study characteristics for the two groups of included studies.

### Findings as reported by the study authors

Results for the included studies are summarised in online supplementary table S4. In hospital mortality refers to death during the admission for the procedure. ORs signify the risk of death when different volumes are compared with 95% CIs where reported. Detailed analysis of the results of the 34 included studies is available in the full report.^5^

Studies on single conditions or procedures were more likely to identify an effect of volume on mortality but these focused on high risk conditions and procedures. Even within these highly selected groups there was considerable variation in effect depending on procedure type and individual centre or centre performance. The effect of surgeon volume illustrates this variability. Of four studies that included an examination of the effects of surgeon volume as well as centre volume, two found an association of decreasing mortality with increasing surgeon volume,^35^ ^41^ one found increasing surgeon volume decreased mortality for only one of four complex conditions^36^ and one study found no association between surgeon volume and outcome.^31^

The findings from studies that included broader CHD populations were more equivocal. In some studies where an effect was identified, the effect was weak or only demonstrable for specific subgroups of patients. There was no clear indication that the evidence for the volume and mortality relationship was substantially stronger than the evidence for a no effect relationship in this broader group of patients Two large, comprehensively adjusted studies showed that, while a volume relationship exists, effects are small in comparison to factors such as condition severity and associated surgical risk, and surgical era.^23^ ^26^

Overall, the evidence does demonstrate a relationship between volume and outcome in the majority of studies, although this relationship is not consistent. While volume is an important factor to consider the evidence highlights the complex relationship between volume, outcome and other factors which may also have an effect.

## Discussion

This review found a substantial body of evidence reporting a positive relationship between volume and outcome, particularly for highly complex cases. However, interpretation is not straightforward. The 34 included studies revealed considerable variability highlighting the complexity of this relationship, as well as identifying variation in methods and findings across individual studies, and the methodological limitations imposed by the research approaches taken. Interpreting the evidence is particularly challenging due to a lack of information on clinical and service-related processes in the literature making it difficult to disentangle the volume/outcome relationship from other clinical and service processes and outcomes.

We have identified five key findings relevant to the organisation and delivery of CHD specialist services.

First, a range of factors influence mortality in CHD of which centre volume is only one**.** Our data extraction identified 67 different variables used to adjust for risk in the included studies, the most influential being condition severity.

Second, the included studies show that clinical advances, increasing expertise and changes in service provision have also influenced and improved outcomes for CHD over time. Five studies that analysed data over periods spanning up to 10 years found that, irrespective of other factors including volume and despite increasing complexity, mortality decreased over the study period. Therefore, the relevance of findings from historical data to contemporary services needs to be carefully considered.

Third, many studies used aggregated data from a large number of centres. Although this approach may show a difference in mortality rates between high and low volume centres, it may mask between-centre variation**.** Several included studies identified this variation with some low or medium volume centres performing as well as those with high volume indicating that individual centre effects are also likely to influence outcomes.

Fourth, the available evidence on the relationship between volume and mortality is dominated by retrospective studies conducted within the USA. It includes centres with very small volumes of cases limiting generalisability. The organisation of services in the USA is very different to the UK and other countries where centralisation of CHD services has led to a consequent increase in volume as more cases become concentrated in fewer centres. It remains unclear whether the impact of volume on outcome is largely a consequence of higher volume units organising and providing a complex service with all the ‘right’ components, is an independent factor directly related to the advantages of dealing with a larger number of cases or a combination of both. The lack of any UK studies to contribute to the review indicates a serious gap in evidence relevant to NHS service provision.

Finally, few studies are able to suggest an optimal size of a CHD centre in terms of volume. Less than half of the included studies analysed volume as a continuous variable which would provide the most robust evidence from which to consider volume thresholds.

The effects of some factors, such as condition severity, are well established but the effect of processes, systems and individual clinician effects on outcome remain unknown.

The full review also included evidence from three studies on adult CHD. One included heart transplant patients for a range of conditions in addition to CHD and so was of limited value.^28^ Two studies explored the effect of surgeon type in relation to outcome.^15^ ^18^ Both studies found adult patients with CHD had better outcomes when operated on by paediatric surgeons in specialist children's centres.

### Strengths and weaknesses of this study

This review was commissioned to inform an ongoing service review and was completed within 3 months. Rapid reviews have evolved primarily to inform emergent decision-making in healthcare settings.^42^ The short time frame and streamlined methodology that they utilise require a compromise between the need for efficiency against exhaustive evidence identification and synthesis. An examination of recent rapid reviews found considerable variation in the methodologies adopted and acknowledges that there is not a ‘one size fits all’ approach. Methods used should therefore be clear and transparent.^43^ The key strengths of our approach are clear and reproducible methods for evidence searches; inclusion and exclusion criteria to identify relevant evidence and structured data extraction.

Time constraints meant we did not search exhaustively but aimed to identify all key evidence of relevance. It is possible that we may have missed relevant evidence. However, we did conduct citation searches on all included studies to minimise the likelihood of omitting eligible studies. Data extraction focused on identifying critical information for evidence synthesis rather than exhaustively extracting and critiquing all available information within individual papers. We were only able to conduct limited checking for screening and data extraction. A second reviewer screened 10% of the references identified from the searches. Data extraction was undertaken by five reviewers with but double data extraction was undertaken for a sample of included papers to refine the data extraction form and queries about data extraction or inclusions were resolved by discussion within the review team.

A meta-analysis of the evidence on volume and outcome was judged to be of limited value given the identified heterogeneity of context and populations. Further review of the broader fields of cardiac surgery outside CHD could contribute to identifying clinical and service-related processes and outcomes that may be relevant and provide a framework for future data collection.

Instead of conducting a detailed quality appraisal of individual studies, we examined study methodology and generic study design limitations, including self-reported generic limitations, to construct a collective assessment of study quality.

### Strengths and weaknesses of included studies

Information bias might result from missing data, miscoding or misinterpretation of information provided in routine databases. Several studies included in this review cited incomplete data as an issue,^35^ ^39^ ^25^ ^27^ for example, missing surgeon identifiers,^15^ limited exploration of the surgeon volume and outcome relationship. Some data sources relied on voluntary completion^23^ ^36^ ^40^ which introduces potential selection bias through coverage, membership or criteria for case submission.^34^ ^36^ Inconsistency in coding, particularly over time, can lead to errors and routine databases may not include information on important contextual details about individual institutions such as team composition, training and experience, type of facility and access to specialist facilities, services and care pathways. Critical details such as non-intervention, transfers between institutions and preoperative mortality are frequently not recorded. This lack of information means the ability to assess the impact of other aspects of care will remain constrained.

Data relating to a single institution is unlikely to be generalisable. Analysing data from a single year overcomes some of the confounding effects related to structural or process changes over time and the associated danger that results measured at different time points may be misinterpreted. Study reports of a single surgical procedure can produce valuable insights for a discrete area of surgical practice but these usually involve rare and complex conditions and small numbers. This combined with the decreasing mortality reducing power, particularly as surgical procedures improve, limits the value of the reported results.

Included studies illustrate significant advances in methodology and analytic approaches over the time period covered by this review.^24^ Increasingly sophisticated tools to score for condition complexity and associated risk of mortality are being developed and methods for handling data as continuous, rather than a categorical, variables is now considered essential. The predominant method of using a step-wise volume category approach to establish a threshold for change in outcome used in many of the included studies is frequently criticised for being unsophisticated and misleading.

### Implications for future research

Our review reveals a clear evidence gap in understanding the relationships between organisational factors in CHD services, how these can potentially predict a range of outcomes relevant to patients and their families, and the causal pathways between organisational factors and outcomes. Better understanding of these relationships is key to the development of methods for assessing and monitoring surgical performance that are not based solely on volume and mortality rates^3^ While existing databases have value in helping understand some relationships and can help inform policy decisions there is scope to develop more comprehensive, high quality clinical and administrative databases to collect information on a range of organisational factors and outcomes related to quality of care. In the UK there is scope to expand the existing National Institute for Cardiovascular Outcomes Research (NICOR) database to capture more of this information. A more sophisticated information resource could then be used to conduct high quality studies of the relationship between organisational factors, volume and outcomes of direct relevance to the NHS and to improve the evidence base to support decisions about the organisation and delivery of CHD services.

## Conclusion

This attempt to locate intervention or observational studies on the relationship between volume and other related organisational features and patient outcomes for adults and children with CHD identified a substantial volume of studies. Observational studies reported the relationship between volume and outcome in congenital heart services, particularly for paediatric surgery. This extensive body of evidence reveals a range of factors, in addition to volume, that influence outcome. These include condition severity, individual centre and surgeon effects and clinical advances over time. The heterogeneity of findings from observational studies suggests that, while a relationship between volume and outcome exists, this is unlikely to be a simple, independent and directly causal relationship. The effect of volume on outcome relative to the effect of other as yet undetermined health system factors remains a complex and unresolved research question.

## References

[1] Luft HS The relation between surgical volume and mortality: an exploration of causal factors and alternative models. Med Care 1980;18:940–59. 10.1097/00005650-198009000-00006 7432019

[2] Pieper D, Mathes T, Neugebauer E State of evidence on the relationship between high-volume hospitals and outcomes in surgery: a systematic review of systematic reviews. J Am Coll Surg 2013;216:1015–25. 10.1016/j.jamcollsurg.2012.12.049 23528183

[3] Kalfa D, Chai P, Bacha E Surgical volume-to-outcome relationship and monitoring of technical performance in pediatric cardiac surgery. Pediatr Cardiol 2014;35:899–905. 10.1007/s00246-014-0938-y 24894896

[4] NHS Specialised Services. Review of Children's Cardiac Services in England: Decision Making Business Case. London: NHS Specialised Services, 2012. http://www.webarchive.org.uk/wayback/archive/20130328000255/http://www.specialisedservices.nhs.uk/safe_sustainable/childrens-congenital-cardiac-services

[5] Turner J, Preston L, Booth A What evidence is there for a relationship between organisational features and patient outcomes in congenital heart disease services? A rapid review. Health Serv Deliv Res 2014;2:1–120. 10.3310/hsdr02430 25642567

[6] Papaioannou D, Sutton A, Carroll C Literature searching for social science systematic reviews: consideration of a range of search techniques. Health Info Libr J 2010;27:114–22. 10.1111/j.1471-1842.2009.00863.x 20565552

[7] Moher D, Liberati A, Tetzlaff J, et al., The PRISMA Group. Preferred reporting items for systematic reviews and meta-analyses: the PRISMA Statement. PLoS Med 2009;6:e1000097 10.1371/journal.pmed.1000097 19621072 PMC2707599

[8] Arenz C, Asfour B, Hraska V Congenital heart surgery: surgical performance according to the Aristotle complexity score. Eur J Cardiothorac Surg 2011;39:e33–7. 10.1016/j.ejcts.2010.11.062 21232971

[9] Bazzani LG, Marcin JP Case volume and mortality in pediatric cardiac surgery patients in California, 1998–2003. Circulation 2007;115:2652–9. 10.1161/CIRCULATIONAHA.106.678904 17485577

[10] Benavidez OJ, Gauvreau K, Del Nido P Complications and risk factors for mortality during congenital heart surgery admissions. Ann Thorac Surg 2007;84:147–55. 10.1016/j.athoracsur.2007.02.048 17588402

[11] Chang RK, Rodriguez S, Lee M Risk factors for deaths occurring within 30 days and 1 year after hospital discharge for cardiac surgery among pediatric patients. Am Heart J 2006;152:386–93. 10.1016/j.ahj.2005.12.016 16875927

[12] Dinh K, Maroulas V Statistical modelling of mortality risk for congenital heart defects. J Appl Quantit Methods 2010;5:670–8.

[13] Gray DT, Louhimo I, Ahonen J Inter-institutional variation in risk-adjusted paediatric cardiac surgical outcomes. Prog Pediatr Cardiol 2003;18:33–42. 10.1016/S1058-9813(03)00074-2

[14] Hickey P, Gauvreau K, Connor J The relationship of nurse staffing, skill mix, and Magnet recognition to institutional volume and mortality for congenital heart surgery. J Nurs Admin 2010;40:226–32. 10.1097/NNA.0b013e3181da3f71 20431457

[15] Karamlou T, Diggs BS, Person T National practice patterns for management of adult congenital heart disease: operation by pediatric heart surgeons decreases in-hospital death. Circulation 2008;118:2345–52. 10.1161/CIRCULATIONAHA.108.776963 18997167

[16] Kazui T, Osada H, Fujita H, Committee for Scientific Affairs. An attempt to analyze the relation between hospital surgical volume and clinical outcome. Gen Thorac Cardiovasc Surg 2007;55:483–92. 10.1007/s11748-007-0172-0 18066639

[17] Mery CM, Moffett BS, Khan MS Incidence and treatment of chylothorax after cardiac surgery in children: analysis of a large multi-institution database. J Thorac Cardiovasc Surg 2014;147:678–86. 10.1016/j.jtcvs.2013.09.068 24246545

[18] Kim YY, Gauvreau K, Bacha EA Risk factors for death after adult congenital heart surgery in pediatric hospitals. Circ Cardiovasc Qual Outcomes 2011;4:433–9. 10.1161/CIRCOUTCOMES.110.958256 21693722

[19] Oster ME, Strickland MJ, Mahle WT Impact of prior hospital mortality versus surgical volume on mortality following surgery for congenital heart disease. J Thorac Cardiovasc Surg 2011;142:882–6. 10.1016/j.jtcvs.2011.04.011 21571324

[20] Pasquali SK, Li JS, Burstein DS Association of center volume with mortality and complications in pediatric heart surgery. Pediatrics 2012;129:e370–6. 10.1542/peds.2011-1188 22232310 PMC3269112

[21] Sakata R, Kuwano H, Yokomise H Hospital volume and outcomes of cardiothoracic surgery in Japan: 2005–9 national survey. Gen Thorac Cardiovasc Surg 2012;60:625–38. 10.1007/s11748-012-0128-x 22907200

[22] Seifert HA, Howard DL, Silber JH Female gender increases the risk of death during hospitalization for pediatric cardiac surgery. J Thorac Cardiovasc Surg 2007;133:668–75. 10.1016/j.jtcvs.2006.11.014 17320563

[23] Vinocur JM, Menk JS, Connett J Surgical volume and center effects on early mortality after pediatric cardiac surgery: 25-year North American experience from a multi-institutional registry. Pediatr Cardiol 2013;34:1226–36. 10.1007/s00246-013-0633-4 23377381 PMC4357309

[24] Welke KF, Shen I, Ungerleider RM Current assessment of mortality rates in congenital cardiac surgery. Ann Thorac Surg 2006;82:164–70. 10.1016/j.athoracsur.2006.03.004 16798208

[25] Welke KF, Diggs BS, Karamlou T The relationship between hospital surgical case volumes and mortality rates in pediatric cardiac surgery: a national sample, 1988–2005. Ann Thorac Surg 2008;86:889–96. 10.1016/j.athoracsur.2008.04.077 18721578

[26] Welke KF, O'Brien SM, Peterson ED The complex relationship between pediatric cardiac surgical case volumes and mortality rates in a national clinical database. J Thorac Cardiovasc Surg 2009;137:1133–40. 10.1016/j.jtcvs.2008.12.012 19379979

[27] Welke KF, Karamlou T, Ungerleider RM Mortality rate is not a valid indicator of quality differences between pediatric cardiac surgical programs. Ann Thorac Surg 2010;89:139–44. 10.1016/j.athoracsur.2009.08.058 20103224

[28] Arnaoutakis GJ, George TJ, Allen JG Institutional volume and the effect of recipient risk on short-term mortality after orthotopic heart transplant. J Thorac Cardiovasc Surg 2012;143:157–67. 10.1016/j.jtcvs.2011.09.040 22172752 PMC4127199

[29] Berry JG, Cowley CG, Hoff CJ In-hospital mortality for children with hypoplastic left heart syndrome after stage I surgical palliation: teaching versus nonteaching hospitals. Pediatrics 2006;117:1307–13. 10.1542/peds.2005-1544 16585328

[30] Berry JG, Lieu TA, Forbes PW Hospital volumes for common pediatric specialty operations. Arch Pediatr Adolesc Med 2007;161:38–43. 10.1001/archpedi.161.1.38 17199065

[31] Checchia PA, McCollegan J, Daher N The effect of surgical case volume on outcome after the Norwood procedure. J Thorac Cardiovasc Surg 2005;129:754–9. 10.1016/j.jtcvs.2004.07.056 15821640

[32] Davies RR, Russo MJ, Hong KN Increased short- and long-term mortality at low-volume pediatric heart transplant centers: should minimum standards be set? Retrospective data analysis. Ann Surg 2011;253:393–401. 10.1097/SLA.0b013e31820700cc 21183849

[33] Dean PN, McHugh KE Conaway MR Effects of race, ethnicity, and sex on surgical mortality in hypoplastic left heart syndrome. Pediatr Cardiol 2013;34:1829–36. 10.1007/s00246-013-0723-3 23722968 PMC4023351

[34] Hirsch JC, Gurney JG, Donohue JE Hospital mortality for Norwood and arterial switch operations as a function of institutional volume. Pediatr Cardiol 2008;29:713–17. 10.1007/s00246-007-9171-2 18080151

[35] Hornik CP, He X, Jacobs JP Relative impact of surgeon and center volume on early mortality after the Norwood operation. Ann Thorac Surg 2012;93:1992–7. 10.1016/j.athoracsur.2012.01.107 22516833 PMC3469698

[36] Karamlou T, McCrindle BW, Blackstone EH Lesion-specific outcomes in neonates undergoing congenital heart surgery are related predominantly to patient and management factors rather than institution or surgeon experience: a Congenital Heart Surgeons Society Study. J Thorac Cardiovasc Surg 2010;139:569–77. 10.1016/j.jtcvs.2008.11.073 19909989

[37] McHugh KE, Hillman DG, Gurka MJ Three-stage palliation of hypoplastic left heart syndrome in the University HealthSystem Consortium. Congenital Heart Dis 2010;5:8–15. 10.1111/j.1747-0803.2009.00367.x 20136852

[38] Morales DL, Zafar F, Rossano JW Use of ventricular assist devices in children across the United States: analysis of 7.5 million pediatric hospitalizations. Ann Thorac Surg 2010;90:1313–18. 10.1016/j.athoracsur.2010.04.107 20868835

[39] Pasquali SK, Jacobs JP, He X The complex relationship between center volume and outcome in patients undergoing the Norwood operation. Ann Thorac Surg 2012;93:1556–62. 10.1016/j.athoracsur.2011.07.081 22014746 PMC3334400

[40] Petrucci O, O'Brien SM, Jacobs ML Risk factors for mortality and morbidity after the neonatal Blalock–Taussig shunt procedure. Ann Thorac Surg 2011;92:642–51. 10.1016/j.athoracsur.2011.02.030 21550583

[41] Tabbutt S, Ghanayem N, Ravishankar C Risk factors for hospital morbidity and mortality after the Norwood procedure: a report from the Pediatric Heart Network Single Ventricle Reconstruction trial. J Thorac Cardiovasc Surg 2012;144:882–95. 10.1016/j.jtcvs.2012.05.019 22704284 PMC4385520

[42] Khangura S, Konnyu K, Cushman R Evidence summaries: the evolution of a rapid review approach. Syst Rev 2012;1:10 10.1186/2046-4053-1-10 22587960 PMC3351736

[43] Harker J, Kleijnen J What is a rapid review? A methodological exploration of rapid reviews in health technology assessments. Int J Evid Based Healthc 2012;10:397–410. 10.1111/j.1744-1609.2012.00290.x 23173665

